# Transfection of Vitamin D3-Induced Tolerogenic Dendritic Cells for the Silencing of Potential Tolerogenic Genes. Identification of CSF1R-CSF1 Signaling as a Glycolytic Regulator

**DOI:** 10.3390/ijms22147363

**Published:** 2021-07-08

**Authors:** María José Mansilla, Iñigo González-Larreategui, Neus Figa-Martín, Jaume Barallat, Federico Fondelli, Ares Sellés-Rius, Bibiana Quirant-Sánchez, Aina Teniente-Serra, Eva Martínez-Cáceres

**Affiliations:** 1 Division of Immunology, Germans Trias i Pujol University Hospital and Research Institute, Campus Can Ruti, 08916 Badalona, Spain; mjmansilla@igtp.cat (M.J.M.); inigogonzalezlarreategui@gmail.com (I.G.-L.); neusfiga@gmail.com (N.F.-M.); ffondelli@igtp.cat (F.F.); aselles@igtp.cat (A.S.-R.); bquirant.germanstrias@gencat.cat (B.Q.-S.); ateniente@igtp.cat (A.T.-S.); 2 Department of Cellular Biology, Physiology and Immunology, Universitat Autònoma de Barcelona, 08193 Bellaterra, Spain; 3 Department of Biochemistry, Germans Trias i Pujol University Hospital, 08916 Badalona, Spain; jbarallat.germanstrias@gencat.cat

**Keywords:** tolerance, autoimmunity, vitamin D3, tolerogenic dendritic cells, CSF1R, DC-SIGN

## Abstract

The use of autologous tolerogenic dendritic cells (tolDC) has become a promising strategy to re-establish immune tolerance in autoimmune diseases. Among the different strategies available, the use of vitamin D3 for the generation of tolDC (VitD3-tolDC) has been widely tested because of their immune regulatory properties. To identify molecules and pathways involved in the generation of VitD3-tolDC, we established an easy and fast gene silencing method based on the use of Viromer blue to introduce siRNA into monocytes on day 1 of culture differentiation. The analysis of the effect of *CD209* (DC-SIGN) and *CD115* (CSF1R) down-modulation on the phenotype and functionality of transfected VitD3-tolDC revealed a partial role of CD115 in their tolerogenicity. Further investigations showed that CSF1R-CSF1 signaling is involved in the induction of cell metabolic reprogramming, triggering glycolysis to produce high amounts of lactate, a novel suppressive mechanism of T cell proliferation, recently found in autologous tolerogenic dendritic cells (ATDCs).

## 1. Introduction

Since the beginning of the 21st century, researchers have focused their efforts on using dendritic cells (DC) as a powerful tool to control altered immune responses and restore the homeostatic balance. DC are a heterogeneous group of potent antigen-presenting cells (APC) with the capacity of processing and presenting antigens to naïve T cells, thus priming adaptive immune response [1]. Importantly, DC are able to control T cell activation, making them a promising treatment not only for cancer therapy, but also for restoring immune tolerance in autoimmune diseases and avoiding allograft rejection [2,3].

In this context, tolerogenic dendritic cells (tolDC) represent a recent emerging strategy to induce a specific and long-term re-establishment of the immune tolerance towards self-antigens causing the immunogenic response of autoimmune diseases such as multiple sclerosis (MS), rheumatoid arthritis (RA) or type-1 diabetes (T1D). In this way, a tolDC strategy avoids the chronic and general immunosuppression of current treatments producing different serious adverse effects in patients [4].

TolDC are defined as semi-mature DC, an intermediate state of maturation between immature DC (iDC) and mature DC (mDC), combining characteristics of both states. Phenotypically, tolDC express low levels of co-stimulatory molecules (CD80, CD83, CD86) and HLA-DR compared to mDC. Functionally, tolDC secrete anti-inflammatory cytokines (IL-10, TGF-β) and have reduced capacity to trigger T cell proliferation due to the induction of regulatory T cells (Treg), T cell anergy/depletion or T cell hyporesponsiveness [5,6,7,8]. Importantly, in contrast to iDC, tolDC are phenotypically stable and upon pro-inflammatory stimuli they do not become mDC. Therefore, tolDC are not potentially harmful when being injected in patients with an inflammatory disease.

DC can be derived ex vivo from autologous peripheral blood monocytes in the presence of granulocyte-macrophage colony-stimulating factor (GM-CSF) and IL4 [9], and different protocols have been described to generate tolDC by using anti-inflammatory cytokines (IL-10, TGF-β), pharmacological agents (vitamin D3, dexamethasone, rapamycin), commercialized drugs (aspirin, simvastatin) or by genetic engineering [5,10,11,12]. From all these protocols, the use of the active form of vitamin D3 (VitD3, 1,25-dyhydroxyvitamin D3) is one of the approaches most widely used to generate tolDC due to their reported characteristics: VitD3-tolDC show a semi-mature phenotype and can induce CD4^+^ Foxp3^+^ Treg, T cell hyporesponsiveness and a switch of the immune response towards a Th2 profile [10,13,14].

To date, several phase I clinical trials have been conducted or are currently ongoing using tolDC in patients with RA, MS, optic neuromyelitis, Crohn’s disease and T1D [4,15]. All these studies indicate that tolDC therapies are safe, do not present relevant side effects and are well tolerated by patients. However, in-depth characterization of tolDC is still required to improve their effectiveness and their translation into the clinic.

To date, no universal tolDC biomarker has been identified [16]. Currently, quality control studies to evaluate the proper tolDC production prior to their administration into the patient are based on phenotyping and/or allogeneic T cell proliferation assays, which are unreliable or time-consuming methods, respectively. An appropriate selection of biomarkers of tolerogenicity would allow us to guarantee the therapeutical success and biosafety of these treatments by providing a faster and more reliable way of validating the effectiveness of the generated cellular product.

To improve the efficacy of VitD3-tolDC therapy for future phase II clinical trials, we have been investigating transcriptional differences between mDC and VitD3-tolDC to identify the molecules and pathways involved in tolerogenicity. Through a microarray analysis we found upregulated in VitD3-tolDC, compared to mDC, the genes *CD115* (or *CSF1R*, that encodes for macrophage colony-stimulating factor receptor) as well as *CSF1* (colony-stimulating factor 1, also known as macrophage colony-stimulating factor, M-CSF, one of the two possible CSF1R ligands), among others [17]. It has been reported that CSF1R regulates macrophage survival and differentiation [18] and, interestingly, in the presence of IL-4 CSF1-CSF1R signaling promotes the differentiation of CD14⁺ monocytes into suppressive macrophages that inhibit CD8^+^ T cell proliferation and expand CD4^+^ Foxp3^+^ Treg in vitro [19]. Consistently, CSF1 blockade prevents the in vivo expansion of CD4^+^ Foxp3^+^ Treg cells and abrogates the induction of transplantation tolerance [19]. Indeed, pharmacological CSF1R inhibition is under investigation as a therapeutic strategy for cancer therapy application [20,21,22].

On the other hand, our microarray study also revealed *CD209* (or *DC-SIGN*, which encodes for DC-specific intracellular adhesion molecule 3-grabbing non-integrin) as another upregulated gene in VitD3-tolDC. It has been reported that DC-SIGN has a role in the regulation of T cell proliferation through its interaction with ICAM-3 (intercellular adhesion molecule-3), expressed by T cells [23]. Moreover, DC-SIGN expression is induced by CSF1 + IL-4 and is required for the development of the above-mentioned suppressive capacity of macrophages through IL-10 production [19,24]. All these findings suggest a possible role of CD115 and CD209 molecules in the tolerogenic potential of VitD3-tolDC.

Gene silencing using small interfering ribonucleic acids (siRNA) is a powerful and widely used method to investigate the functional relevance of specific genes. However, strategies available to deliver specific siRNA into cells are not effective for all cell types and require optimization. In this regard, primary human monocyte-derived DC are cells which are difficult to transfect without inducing cell maturation. Some studies have reported optimized protocols for the transfection of monocyte-derived DC [25,26,27]. However, none of them have been optimized so far to deliver siRNA into tolDC during their process of differentiation.

In this study, two different approaches, a lipid-based (HiPerFect^®^) method and a polymer mimicking influenza hemagglutinin virus (Viromer^®^ blue, ViroB) transfection were optimized and tested to downregulate the expression of CD115 and CD209 molecules during the differentiation process of VitD3-tolDC to determine their role in tolerogenicity. However, only ViroB was shown to be a fast and easy strategy to deliver siRNA into monocytes on day 1 of culture without altering viability, phenotype and functionality of VitD3-tolDC.

The application of the ViroB protocol to analyze the role of *CD115* and *CD209* genes revealed that the molecule CSF1R (CD115), but not DC-SIGN (CD209), plays a partial role in the induction of tolerance through VitD3 in tolDC by enhancing glycolysis and glucose consumption to produce lactate, a novel suppressor mechanism of T cell proliferation recently found in autologous tolerogenic dendritic cells (ATDCs) [28].

## 2. Results

### 2.1. HiPerFect Reagent Allows VitD3-tolDC Transfection after Maturation Stimulus

**Figure 1:**
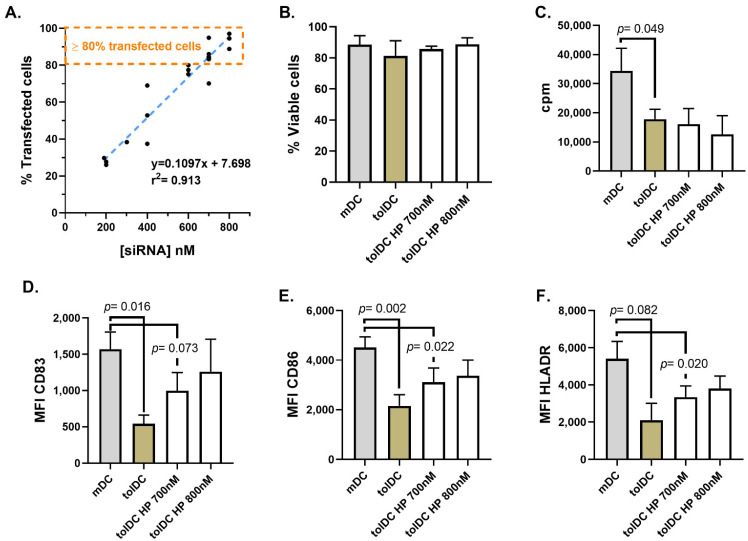
HiPerfect (HP) transfection of monocytes after maturation stimulus using 700 nM and 800 nM of siRNA. Linear regression between the percentage of transfected cells and the concentration of red-labeled non-targeting siRNA (siGLO, negative and red-labeled siRNA control) used (**A**). Characterization of control untreated mDC and VitD3-tolDC, and VitD3-tolDC obtained following HP transfection using 700 nM and 800 nM of siRNA by analyzing their viability (**B**), functionality (ability to induce allogeneic PBMC proliferation using a ratio of DC/PBMC of 1/20) (**C**), and phenotype (mean fluorescence intensity, MFI, of CD83 (**D**), CD86 (**E**) and HLA-DR (**F**) molecules). Data shown as mean + SEM. For all group *n* = 4, except for tolDC HP 800 nM in which *n* = 3.

To investigate the tolerogenic role of potential genes during VitD3-tolDC generation using siRNA technology, the reverse protocol described by Troegeler and colleagues [29] using HiPerFect reagent to transfect monocytes during VitD3-tolDC differentiation was followed. To optimize the transfection of 1 million cells on day 5 of culture (after adding a maturation stimulus), different concentrations of red-labeled non-targeting siRNA (siGLO) were encapsulated with HiPerfect reagent (200 nM, 300 nM, 400 nM, 600 nM, 700 nM and 800 nM of siGLO) and the percentage of red-positive cells was assessed after 36 h by flow cytometry to determine the transfection efficiency. A linear correlation between the concentration of encapsulated control siRNA and the percentage of transfection was found, achieving >80% of cell transfection using 700 nM and 800 nM of siRNA in the wells (700 nM siRNA: 83.83 ± 7.96%, and 800 nM encapsulated siRNA: 93.43 ± 4.20%) (Figure 1A). Importantly, viability, as well as functional and phenotype characteristics of VitD3-tolDC, remained unaltered when cells were transfected with both concentrations of siRNA (Figure 1B–D). Based on the data collected, 700 nM of encapsulated red-labeled non-targeting siRNA was selected as the lowest siRNA concentration appropriated for VitD3-tolDC transfection. However, when the HiPerFect transfection reagent was used to transfect cells in an earlier state of differentiation, the manipulation of cells before receiving the maturation stimulus (day 4 of culture) activated them, preventing the effect of vitamin D3. In accordance, cells acquired characteristics closer to mDC, compared to untreated VitD3-tolDC (Supplementary Figure S1).

### 2.2. Viromer Blue Allows Monocyte Transfection during Their Differentiation to VitD3-tolDC

**Figure 2:**
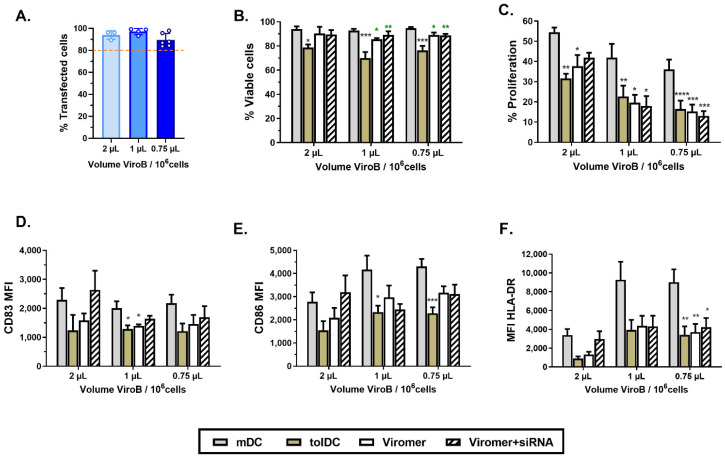
Transfection of monocytes on day 1 of differentiation using Viromer blue. Analysis of transfected (red-labeled or siGLO^+^ cells) using 2 µL, 1 µL and 0.75 µL of Viromer blue (ViroB) (**A**). Characterization of mDC, VitD3-tolDC and VitD3-tolDC obtained following ViroB or ViroB+non-targeting siRNA (ViroB+siRNA) treatment using 2 µL, 1 µL and 0.75 µL of ViroB. Analysis of viability (**B**), functionality (ability to induce allogeneic PBMC proliferation using a ratio of DC/PBMC of 1/20) (**C**), and phenotype (mean fluorescence intensity, MFI, of CD83 (**D**), CD86 (**E**) and HLA-DR (**F**) molecules). Data shown as mean + SEM. For 2 µL, *n* = 3; for 1 µL, *n* = 5; and for 0.75 µL, *n* = 8. Grey and green asterisks represent significance compared to mDC and VitD3-tolDC, respectively (* *p* < 0.05; ** *p* < 0.01; *** *p* < 0.001, ****p < 0.0001).

To achieve an early gene silencing during monocyte differentiation to analyze the functionality of candidate genes, Viromer Blue (ViroB) reagent mixed with or without non-targeting siRNA or siGLO was added on day 1 of culture following the manufacturer’s procedure. To set up the volume of ViroB required to transfect monocytes without altering their differentiation in VitD3-tolDC, 2 µL, 1 µL and 0.75 µL of ViroB complexed with 275 nM siRNA were tested to transfect 1 million of monocytes in 1 mL of medium after 24 h of culture. The transfection efficiency (indicated by the % of siGLO positive cells on day 6 of culture) achieved was >80% in all the experiments and was similar between the three volumes of ViroB used (2 µL: 93.90 ± 3.90%; 1 µL: 97.10 ± 2.81%; and 0.75 µL: 89.40 ± 5.98%) (Figure 2A).

To analyze the toxicity of ViroB during the culture, viability was assessed in mDC, VitD3-tolDC and VitD3-tolDC treated with ViroB (without siRNA) and VitD3-tolDC treated with ViroB + siRNA (non-targeting siRNA) on day 6 of culture (Figure 2B). As expected, the treatment with VitD3 significantly reduced the viability of tolDC compared to mDC (Figure 2B). Importantly, the viability of VitD3-tolDC was not decreased by the addition of ViroB to the medium on day 1 with any of the volumes tested (Figure 2B). However, functional (Figure 2C) and phenotype (Figure 2D–F) analysis revealed that when using 2 µL of ViroB, the characteristics of VitD3-tolDC treated with ViroB with or without siRNA were altered (although due to the low number of samples, statistical differences were not observed compared to control VitD3-tolDC), showing a profile more similar to mDC than untreated VitD3-tolDC (Figure 2C–F). In contrast, in the case of adding 1 µL or 0.75 µL of ViroB to the culture on day 1, phenotype and functional characteristics of VitD3-tolDC cells remained unaltered compared to control VitD3-tolDC (Figure 2C–F). Consequently, 0.75 µL of ViroB was selected as the lowest appropriate volume to perform gene silencing experiments in VitD3-tolDC.

### 2.3. CD209 Gene Silencing in VitD3-tolDC Alters Neither Their Phenotype nor Their Functionality

**Figure 3:**
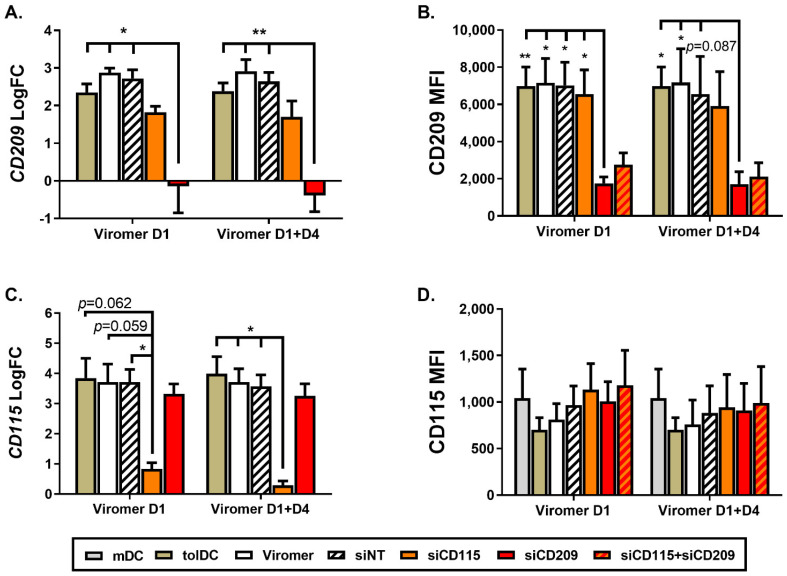
Down-modulation of CD115 and CD209 expression. Analysis of gene and protein expression of CD209 (**A**,**B**) and CD115 (**C**,**D**) by using specific siRNA + ViroB treatment of VitD3-tolDC on day 1 (D1) or on day 1 + day 4 (D1 + D4) of culture differentiation. Data shown as mean + SEM. *n* = 3 for all groups. Data of gene expression are referred to mDC and showed as logarithm in base 2 of fold change (FC). Graph B displays only statistical differences between siCD209 and the rest of the tolDC conditions. MFI: mean fluorescence intensity. * *p* < 0.05; ** *p* < 0.01.

Gene expression analysis on day 6 of culture confirmed results from the microarray study and showed an increase of 3.26 ± 1.61 fold change (FC) in the expression of *CD209* in VitD3-tolDC compared to mDC (*p* = 0.008, *n* = 8), which was also related to an overexpression of CD209 (DC-SIGN) at protein level (CD209 mean fluorescence intensity, MFI = mDC: 21,263 ± 7099 vs. tolDC: 28,462 ± 6865, *p* = 0.018, *n* = 6). To investigate the tolerogenic properties of CD209 molecule, monocytes on day 1 of culture differentiation were transfected with a pool of four specific *CD209* siRNA (siCD209) at 275 nM complexed with 0.75 µL of ViroB per 1 million of cells plated on a 24-well plate. Gene expression analysis on day 6 of culture showed a *CD209* specific inhibition of 84.84 ± 7.21% in VitD3-tolDC treated with ViroB + siCD209 on day 1 of culture compared to VitD3-tolDC treated with non-targeting siRNA (VitD3-tolDC + siNT) (*CD209* logFC = siNT: 2.72 ± 0.39 vs. siCD209: −0.14 ± 1.23, *p* = 0.028) (Figure 3A). Moreover, flow cytometry analysis confirmed the silencing of *CD209* gene and exhibited a 73.27 ± 11.05% reduction of CD209 protein expression in the surface of cells transfected with ViroB + siCD209 on day 6 of culture (mean fluorescence intensity of CD209 = siNT: 7029.33 ± 2157.38 vs. siCD209: 1754.33 ± 585.15, *p* = 0.015) (Figure 3B). In addition, when siCD209 transfection on VitD3-tolDC was performed twice, on day 1 and day 4 of culture, similar results of CD209 gene and protein expression were found: *CD209* gene silencing of 87.53 ± 3.27% (*CD209* logFC = siNT: 2.65 ± 0.41 vs. siCD209: −0.40 ± 0.74, *p* = 0.005) and protein down-modulation of 72.46 ± 11.78% (CD209 MFI = siNT: 6544.67 ± 3522.5 vs. siCD209: 1713.33 ± 1155.73, *p* = 0.087) (Figure 3A,B).

**Figure 4:**
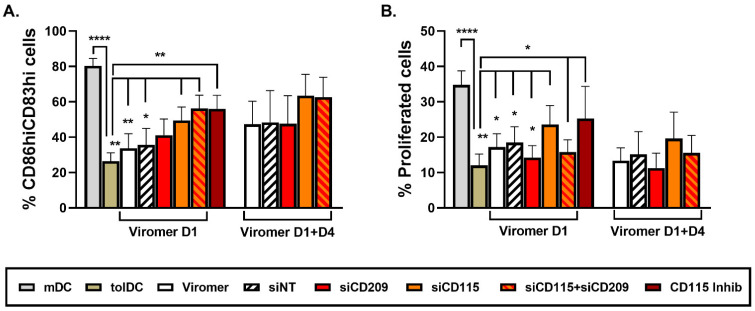
CD115 down-modulation partially alters phenotype and functionality of VitD3-tolDC. Analysis of phenotype (% CD83^hi^ CD86^hi^ cells) (**A**) and functionality (ability to induce allogeneic PBMC proliferation using a ratio of DC/PBMC of 1/20) (**B**) of VitD3-tolDC treated with CD115-siRNA and/or CD209-siRNA on day 1 (D1) or on day 1 + day 4 (D1 + D4) of culture. In addition to controlling transfection conditions (Viromer and siNT), cells also treated on day 0 + day 4 with a specific CD115-inhibitor (PLX5622) were included. Data shown as mean + SEM. For mDC and VitD3-tolDC, *n* = 8; for conditions of transfection with Viromer on D1, *n* = 6; and for conditions of transfection with Viromer on D1 + D4 and for CD115 Inhibitor, *n* = 3. * *p* < 0.05; ** *p* < 0.01; **** *p* < 0.0001.

Results from the phenotypic analysis of *CD209* down-modulated VitD3-tolDC exhibited no modifications in the percentage of CD83^hi^CD86^hi^ cells (Figure 4A). In accordance, the functional analysis did not show alterations in the induction of allogeneic PBMC proliferation by silenced CD209 VitD3-tolDC compared to control siRNA-treated VitD3-tolDC (Figure 4B).

### 2.4. CD115 Gene Silencing in VitD3-tolDC Partially Inhibits Their Tolerogenic Properties

CD115 (CSF1R) molecule was selected as a candidate gene to evaluate its potential role in VitD3-tolDC functionality since an overexpression of 2.71 ± 1.00 FC in VitD3-tolDC compared to mDC (*p* = 0.008, *n* = 8) was observed. Unexpectedly, differences in CD115 expression at protein level between mDC and VitD3-tolDC were not found (CD115 MFI = mDC: 691 ± 227.9 vs. tolDC: 798.5 ± 247.9, *p* = 0.204, *n* = 6) by flow cytometry analysis of its surface expression. Following specific silencing of *CD115* gene by treating VitD3-tolDC with ViroB + *CD115* siRNA (siCD115) on day 1 or on day 1 + day of 4 culture, a gene down-modulation of 82.21 ± 10.99% and 95.16 ± 6.84% of CD115 in siCD115-VitD3-tolDC was achieved, respectively, compared to control siRNA-treated VitD3-tolDC (*CD115* logFC day 1: siNT: 3.71 ± 0.73 vs. siCD115: 0.83 ± 0.36, *p* = 0.034; and *CD115* logFC day 1 + 4: siNT: 3.57 ± 0.67 vs. siCD115: 0.29 ± 0.25, *p* = 0.021) (Figure 3C). In accordance with our previous observation, no differences were found on surface CD115 protein expression between different conditions despite the gene silencing treatment (CD115 MFI day 1: siNT: 965.33 ± 359.22 vs. siCD115: 1132 ± 483.89, *p* = 0.146; and CD115 MFI day 1 + day 4: siNT: 884.67 ± 359.22 vs. siCD115: 942.67 ± 611.93, *p* = 0.456) (Figure 3D).

On the other hand, double transfection of VitD3-tolDC with siCD209 and siCD115 (using 50% of each siRNA) resulted in a reduction of CD209 protein expression similar to the one achieved with single siCD209 transfection although statistical differences with siNT-VitD3-tolDC were not reached (probably due to the low number of samples tested, *n* = 3) (Figure 3B). In the case of CD115, in agreement with previous results, no effects were detected at the protein level (Figure 3D).

When cells were characterized, phenotypical examination revealed that VitD3-tolDC treated with siCD115 on day 1 were partially more similar to mDC than VitD3-tolDC and statistically different to control or siNT-treated VitD3-tolDC (%CD83^hi^ CD86^hi^ = mDC: 80.25 ± 12.06%, tolDC: 26.43 ± 13.28% and siNT: 35.62 ± 22.78% vs. siCD115: 49.45 ± 18.67%; *p* = 0.0006, *p* = 0.005 and *p* = 0.014, respectively) (Figure 4A). Similar results were found with double transfection on day 1 + day 4, but statistical differences were not observed, probably due to the low number of samples of these groups (*n* = 3) (Figure 4A). In contrast to siCD115-VitD3-tolDC, silencing of *CD209* in VitD3-tolDC did not result in phenotype alteration. Interestingly, double transfection with siCD115 and siCD209 showed similar results than single transfection of VitD3-tolDC with siCD115 (%CD83^hi^ CD86^hi^ = siCD115 Viromer D1: 49.45 ± 18.67% vs. siCD115 + siCD209: 56.18 ± 16.82, *p* = 0.443; and siCD115 Viromer D1 + D4: 63.47 ± 20.93% vs. siCD115 + siCD209: 62.63 ± 19.45, *p* = 0.458). In line with phenotype results, functional assays also demonstrated a partial reversion of the tolerogenic function of siCD115-VitD3-tolDC (% of alloproliferation = mDC: 34.78 ± 11.33%, tolDC: 12.02 ± 9.09% and siNT: 18.46 ± 10.92% vs. siCD115: 23.54 ± 13.21%; *p* < 0.0001, *p* = 0.005 and *p* = 0.018, respectively) (Figure 4B). In spite of the differences detected in the phenotype analysis, double transfection of VitD3-tolDC with siCD115 + siCD209 did not result in functional alteration (Figure 4B).

In addition, a CD115 (CSF1R) chemical inhibitor, PLX5622, was added to the culture on day 0 and day 4 of monocyte differentiation into VitD3-tolDC to ensure a fast inhibition of CD115 function and to compare its effects on VitD3-tolDC with data obtained using gene silencing on day 1 of culture. In accordance with the results obtained using siCD115 on day 1, cell signaling blockage of CD115 from day 0 of culture also caused a partial abrogation of VitD3-tolDC characteristics and no differences between both techniques were found (%CD83^hi^ CD86^hi^ cells = siCD115: 49.45 ± 18.67% vs. CD115 Inhibitor: 56.03 ± 13.15%, *p* = 0.607; and % of alloproliferation = siCD115: 23.54 ± 13.21% vs. CD115 Inhibitor: 25.26 ± 15.75%, *p* = 0.867) (Figure 4A,B).

### 2.5. CD115 Signaling Induced by CSF1 Triggers Tolerogenic Properties in Monocytes

**Figure 5:**
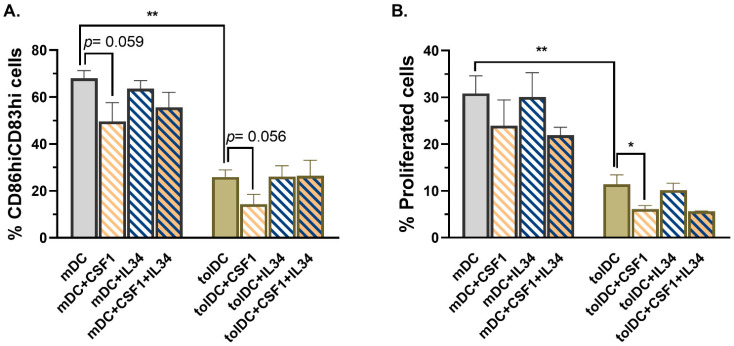
CSF1-CD115 engagement induces tolerogenic properties. Analysis of phenotype (% CD83^hi^ CD86^hi^ cells) (**A**) and functional characteristics (ability to induce allogeneic PBMC proliferation using a ratio of DC/PBMC of 1/20) (**B**) of mDC and VitD3-tolDC exposed to CSF1 and/or IL34 (ligands of CD115) during culture differentiation. Data are shown as mean + SEM. For all groups, *n* = 5, except for mDC + CSF1 + IL34 and tolDC + CSF1 + IL34 in which *n* = 2. * *p* < 0.05; ** *p* < 0.01.

To further analyze the role of CD115 (CSF1R) and its ligands, CSF1 and IL34, mDC and VitD3-tolDC were cultured in the presence of CSF1, IL34 or both during their differentiation (day 0 and 4 of culture). Results obtained revealed that CSF1, but not IL34, increased tolerogenic characteristics of mDC and VitD3-tolDC, although statistical differences were not reached in all the comparisons (% CD83hi CD86hi cells = mDC: 68.02 ± 7.36 vs. mDC + CSF1: 49.56 ± 18.07, *p* = 0.059; and VitD3-tolDC: 25.94 ± 6.74 vs. VitD3-tolDC + CSF1: 14.34 ± 9.45, *p* = 0.056) (Figure 5A) (% of alloproliferation = mDC: 30.86 ± 8.42 vs. mDC + CSF1: 23.94 ± 12.33, *p* = 0.109; and VitD3-tolDC: 11.40 ± 4.57 vs. VitD3-tolDC + CSF1: 6.16 ± 1.63, *p* = 0.029) (Figure 5B). In addition, cells cultured with CSF1 + IL34 did not show an increased tolerogenic phenotype (% CD83hi CD86hi cells = mDC + CSF1: 49.56 ± 18.07 vs. mDC + CSF1 + IL34: 55.70 ± 8.91; and VitD3-tolDC + CSF1: 14.34 ± 9.45 vs. VitD3-tolDC + CSF1 + IL34: 26.40 ± 9.48) (Figure 5A) and functionality was similar but not higher than that found in cells treated only with CSF1 (% of alloproliferation = mDC + CSF1: 23.94 ± 12.33 vs. mDC + CSF1 + IL34: 21.92 ± 2.38; and VitD3-tolDC + CSF1: 6.16 ± 1.63 vs. VitD3-tolDC + CSF1 + IL34: 5.68 ± 0.02) (Figure 5B).

### 2.6. CD115 Induces Glycolysis Activation on VitD3-tolDC

**Figure 6:**
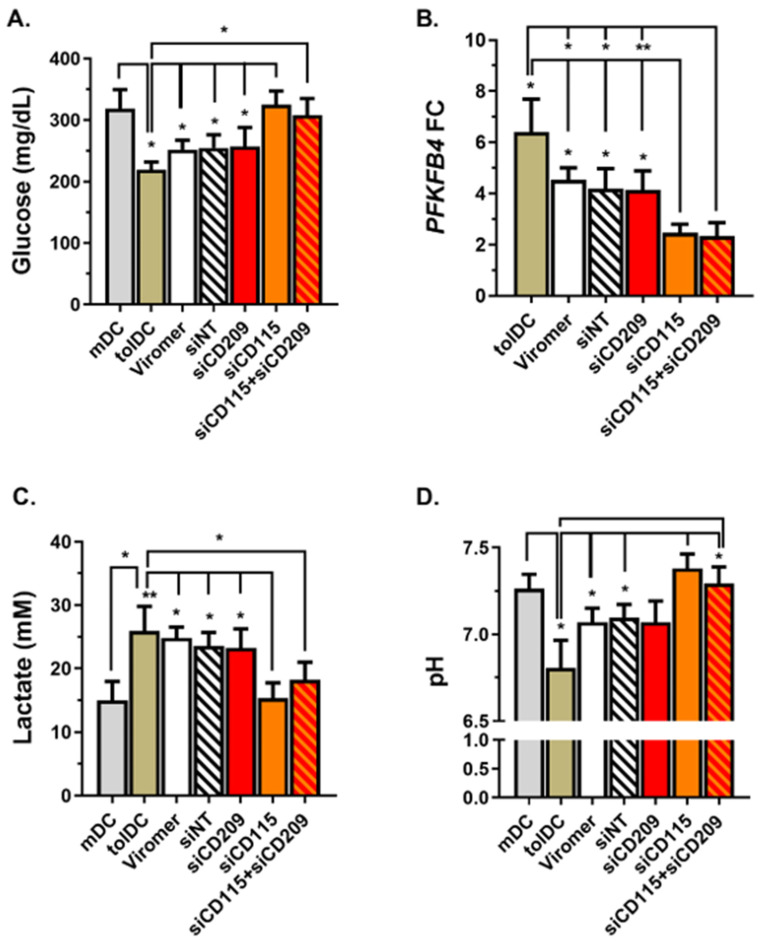
Role of CD115 in metabolism of VitD3-tolDC. Analysis of metabolic characteristics of mDC, VitD3-tolDC and VitD3-tolDC-treated siCD115 and/or siCD209 by determining glucose consumption (**A**), gene expression of 6-Phosphofructo-2-Kinase/Fructose-2,6-Biphosphatase 4 (*PFKFB4*) in cells (**B**), secretion of lactate (**C**) and pH (**D**) of supernatants on day 6 of culture. Data shown as mean + SEM. For glucose concentration, pH and lactate, *n* = 3 (except for mDC and tolDC groups, *n* = 5). For *PFKFB4* gene expression analysis, *n* = 4. * *p* < 0.05; ** *p* < 0.01.

To investigate the mechanisms involved in tolerance induction by CD115 on VitD3-tolDC, glucose consumption was assessed indirectly by determining glucose concentration present in culture supernatants on day 6 of culture. As is shown in Figure 6A, glucose concentration was lower in VitD3-tolDC than in mDC supernatants (219.00 ± 22.07 vs. 318.70 ± 53.38, respectively; *p* = 0.031). Moreover, when CD115 was down-modulated on VitD3-tolDC, glucose consumption was reduced, showing higher glucose levels in the medium compared to control VitD3-tolDC or siNT-VitD3-tolDC (VitD3-tolDC: 219.00 ± 22.07 mg/dL and siNT: 254.70 ± 37.00 mg/dL vs. siCD115: 325.00 ± 38.11 mg/dL, *p* = 0.016 and *p* = 0.023, respectively) (Figure 6A). When concentration of lactate in supernatants and gene expression of 6-Phosphofructo-2-Kinase/Fructose-2,6-Biphosphatase 4 (*PFKFB4*), controls metabolic flux of glycolysis) in cells was examined, an increase of both was found in VitD3-tolDC compared to mDC (lactate concentration = mDC: 15.03 ± 6.54 mmol/L vs. VitD3-tolDC: 25.90 ± 8.73 mmol/L, *p* = 0.037; and *PFKFB4* FC: 6.41 ± 2.56, *p* = 0.035) (Figure 6B,C). Interestingly, *CD115* gene silencing resulted in a partial reversion of lactate secretion level (lactate concentration = VitD3-tolDC: 25.90 ± 8.73 mmol/L and siNT: 23.58 ± 3.66 mmol/L vs. siCD115: 15.33 ± 4.15 mmol/L; *p* = 0.010 and *p* = 0.017, respectively) and a decrease in the expression of *PFKFB4* in VitD3-tolDC (*PFKFB4* FC: VitD3-tolDC: 6.41 ± 2.56 and siNT: 4.19 ± 1.57 vs. siCD115: 2.46 ± 0.67, *p* = 0.048 and *p* = 0.035, respectively), with both more similar to those found in mDC (Figure 6B,C). Finally, pH examination in VitD3-tolDC supernatants showed a reduced pH compared to mDC (7.26 ± 0.15 vs. 6.81 ± 0.27, respectively; *p* = 0.031) in accordance with the yellow tonality of the medium observed in VitD3-tolDC culture medium on day 6. In line with previous results, supernatants of VitD3-tolDC treated with siCD115 exhibited similar pH to the mDC condition (VitD3-tolDC: 7.26 ± 0.15 and siNT: 7.10 ± 0.13 vs. siCD115: 7.38 ± 0.15, *p* = 0.021 and *p* = 0.039, respectively) (Figure 6D).

None of these metabolic-related parameters were found altered in VitD3-tolDC treated with siCD209 (Figure 6). However, double transfection of VitD3-tolDC with siCD115 and siCD209 exhibited the same activation level of *PFKFB4* gene as siCD115-VitD3-tolDC and similar concentrations of glucose and lactate, as well as pH in culture supernatants (Figure 6), although statistical differences were reached only for *PFKFB4* gene expression in siCD115 + siCD209 compared to the VitD3-tolDC and siNT control groups (*PFKFB4* FC: VitD3-tolDC: 6.41 ± 2.56 and siNT: 4.19 ± 1.57 vs. siCD115 + siCD209: 2.34 ± 1.04, *p* = 0.043 and *p* = 0.014, respectively) (Figure 6B).

## 3. Discussion

TolDC cell-based therapy is a promising strategy to restore immune tolerance in autoimmune diseases such as MS. Many in vitro and in vivo studies have demonstrated their ability to control autoreactive T cell proliferation by different mechanisms (induction of Treg and Breg, IL-10 and TGF-β secretion, induction of T cell hyporesponsiveness, among others), as well as their beneficial effect in reducing inflammation and clinical signs when administrated in vivo in animal models. In addition, some phase I clinical trials have reported their safety and good tolerability in RA, T1D, MS and Crohn’s patients [4].

Unfortunately, to date, there is not a universal biomarker for tolDC. Therefore, currently tolDC can be characterized phenotypically, but levels of expression of co-stimulatory molecules and HLA-DR are difficult to standardize and are sometimes unreliable. On the other hand, functional assays are trustworthy but time-consuming because they require around 5 days of co-culture. Consequently, there is an unmet need to find fast and robust functional biomarkers of tolDC to improve their efficacy and translation into the clinic.

In this context, our group has developed an autologous antigen-specific cell therapy based on autologous antigen-specific VitD3-tolDC, which is currently being administrated to active MS patients from a phase I clinical trial (TOLERVIT-MS: Tolerogenic Dendritic Cells as a Therapeutic Strategy for the Treatment of Multiple Sclerosis Patients, EudraCT: 2015-003541-26, ClinicalTrials.gov ID: NCT02903537). To go a step further and try to improve the efficacy of VitD3-tolDC therapy prior to initiating a phase II clinical trial in MS patients, our group has searched for genes differentially expressed in VitD3-tolDC compared to mDC with the objective to identify molecules and pathways involved in the tolerogenicity of VitD3-tolDC.

To investigate the tolerogenic role of the selected differentially expressed genes in VitD3-tolDC, a fast and easy technique is needed. Since most of genes were upregulated in VitD3-tolDC, gene silencing using siRNA was chosen as the method to analyze the effects of loss-of-function of those genes in VitD3-tolDC. Although there are different transfection strategies to introduce siRNA into primary cells (viral vectors, electroporation and lipid-based vectors), monocytes and DC are highly sensitive and especially difficult to transfect. Over recent years, several studies have reported optimized protocols to transfect DC [25,26,27], and tolDC have been generated even by genetic inhibition of co-stimulatory molecules expression [30]. However, these protocols are aimed at transfecting already differentiated DC, but not monocytes during their differentiation culture to tolDC.

In this study, we optimized two different transfection methods, a lipid-based (HiPerFect) and a polymer-based (ViroB) transfection strategy, to deliver siRNA into VitD3-tolDC with high efficiency (>80% transfected cells) and without altering their viability, phenotype and functional characteristics.

HiPerFect is a lipid transfection reagent based on the formation of siRNA packaging vesicles or liposomes structurally similar to cell or organelles membranes, therefore facilitating the cellular uptake and protecting siRNA from enzymatic degradation during cellular endocytosis [31]. In 2014, Troegeler and colleagues developed a protocol using HiPerfect to achieve gene silencing in primary blood monocytes, DC and macrophages without altering their cell functions [29]. We optimized the reported protocol to achieve a high transfection efficiency in monocytes during their culture differentiation into VitD3-tolDC. Although high concentrations of siRNA were needed to achieve >80% transfected cells (700 nM and 800 nM siRNA), the protocol did not cause relevant viability, phenotypical or functional alterations in monocytes transfected on day 5 of differentiation, after receiving the maturation stimulus (at the end of the differentiation culture). However, we cannot discard other alterations caused by the high concentration of siRNA required for the transfection (700 nM), such as the induction of a virus infection-like response triggering apoptosis and inflammasome response in the VitD3-tolDC. Future studies will be required to address this issue. On the other hand, when the HiPerfect protocol was used to transfect monocytes on day 4 of culture (prior to maturation stimulus), cells were sensitive to the manipulation required during the HiPerfect transfection procedure, preventing the effect of VitD3 and inducing phenotypical and functional characteristics typical of mDC in transfected VitD3-tolDC. Therefore, this protocol does not allow the analysis of the tolerogenic function of potential genes during the differentiation of monocytes into VitD3-tolDC.

In recent years, Viromer has been commercialized and is an alternative transfection method taking advantage of the use of a mix of cationic and anionic polymers combined with the endosomal escape mechanism of influenza virus to deliver siRNA inside cells. One of the reasons why the influenza virus is highly infective is the presence of a surface glycoprotein called hemagglutinin, which is responsible for the fusion of the viral envelope with the endocytic vesicle once exposed to low pH, allowing the exit of the genetic content. Viromer mimics this mechanism by using both cationic and anionic polymers, resulting in a neutrally surface-charged vesicle. When these vesicles are exposed to the low pH of the endosome, they become more hydrophobic and are then able to escape from the endosome and to spread their content in the cytoplasm of the cell [32]. Among the different Viromer reagents available, ViroB is specially designed to deliver siRNA. Although both HiPerFect and ViroB are easy-to-use protocols, ViroB requires less manipulation of cells. Thus, it was possible to optimize the ViroB transfection procedure to deliver siRNA in monocytes at the beginning of their differentiation (day 1 of culture) with high efficiency, using a low concentration of siRNA (275 nM siRNA) and without altering viability and tolerogenic characteristics of VitD3-tolDC. Consequently, we established a gene silencing protocol using ViroB + siRNA to evaluate the tolerogenic function of potential genes during the process of monocyte differentiation into VitD3-tolDC.

A study comparing the transcriptomic profile of mDC and VitD3-tolDC revealed an over-expression of *CD115* and *CD209* genes on VitD3-tolDC that was validated by qPCR, although at the protein level only CD209 was found upregulated. It is likely that CD115 protein expression was not found overexpressed in VitD3-tolDC because after CSF1 engagement to CD115 (CSF1R), the cytokine–receptor complex is internalized and degraded [33], with surface determination not a reliable protein expression value for CD115.

When gene and protein expression of CD209 was down-modulated by using Viro + siRNA on day 1 of culture differentiation, no effect on phenotype and functional characteristics of VitD3-tolDC were observed, indicating that CD209 is not directly involved in the tolerogenicity of VitD3-tolDC. Since the effect of siRNA is transient and functional assays require 5 additional days of co-culture after VitD3-tolDC obtention, to confirm results observed following *CD209* gene silencing on day 1 of culture, monocytes were treated with ViroB + siRNA on day 1 + day 4 to achieve a longer knock-down effect. Interestingly, results were similar when using ViroB + siRNA only on day 1 or on day 1 + day 4, meaning that gene silencing is stable after at least 10 days of treatment, and CD209 or DC-SIGN is not involved in the induction of tolerogenic characteristics of VitD3-tolDC. In contrast, gene silencing of *CD115* on day 1 caused a partial reversion of the tolerogenic properties of VitD3-tolDC. These results were similar when *CD115* down-modulation was performed on day 1 + day 4. In addition, double transfection of VitD3-tolDC with siRNA of *CD115* and *CD209* did not increase the effect of *CD115* down-modulation. At the phenotype level, the effect of double CD115 + CD209 siRNA transfection was comparable to the one observed using only *CD115* siRNA, but double gene transfection did not alter functionality of VitD3-tolDC, probably because the effect of using half of the quantity of siRNA for *CD115* is shorter and was not enough to find effects after 5 days of co-culture assay.

Since gene expression results of *CD115* were not correlated with the protein surface expression of CD115 (CSF1R), VitD3-tolDC treated with a specific CD115 cell signaling inhibitor, PLX5622, were included in the study and compared with VitD3-tolDC treated with *CD115* siRNA. Interestingly, results found using the CD115 inhibitor and *CD115* siRNA in VitD3-tolDC were almost identical, validating the partial role of CD115 in the tolerogenicity of VitD3-tolDC.

In addition to *CD115* (CSF1R) overexpression in VitD3-tolDC, an increase of its ligand *CSF1* was also found upregulated in the transcriptional study of VitD3-tolDC compared to mDC. Recently, IL-34 has been identified as a novel CSF1R ligand [34]. It has been reported that CSF1 and IL-34 promote monocyte survival and differentiation into M2-like macrophages with protumoral and immunoregulatory properties [35]. These M2-like macrophages are characterized by producing high levels of IL-10 and low levels of IL-12 [35]. In addition, the expression of CSF1 in the tumor microenvironment is associated with the recruitment, accumulation and polarization of tumor-associated macrophages [18,20,22]. In this context, elevated levels of CSF1 and expression of CSF1R have been associated with poor prognosis in different cancers such as breast and ovarian cancer and, consequently, different strategies have emerged to neutralize or inhibit the CSF1/CSF1R signaling in several cancers [20,21,22].

To further investigate the mechanism induced by CD115 (CSF1R) in VitD3-tolDC, the role of the two CSF1R ligands, CSF1 and IL-34, was examined. Remarkably, only the incubation of mDC and VitD3-tolDC with CSF1 during their process of differentiation reduced the percentage of CD83^hi^CD86^hi^ cells and alloproliferation in both mDC and VitD3-tolDC, further supporting the idea that CSF1-CSF1R cell signaling is involved in the induction of tolerogenic properties of monocytes. Interestingly, Zhu and colleagues reported in 2002 that the incubation of monocytes with the active form of VitD3 (1,25(OH)_2_D_3_) induced a fast (after 1 h) and strong upregulation and secretion of CSF1, which can act in autocrine fashion, blocking the normal DC differentiation mediated by GM-CSF + IL-4 towards a macrophage-like phenotype [36]. Although IL-34 is also involved in M2-like macrophage differentiation, the expression of IL-34 is reduced compared to CSF1, it has two additional receptors (PTPζ and syndecan-1, CD138) and it is a tissue-restricted ligand of CSF1R required for the development of Langerhans cells and microglia. In contrast, CSF1 can only bind to CSF1R and exerts an ubiquitous immunoregulatory function, thus explaining why CSF1 but not IL-34 could be involved in tolerogenicity of VitD3-tolDC.

Previous studies investigating mechanisms involved in the tolerogenic functions gained by monocytes exposed to VitD3 revealed the relevance of metabolic cell reprogramming, with glucose oxidation and glycolysis essential for the VitD3-tolDC function [37,38]. Indeed, the glycolytic enzyme PFKFB4 has been recently identified as a critical checkpoint and direct transcriptional target of VitD3, which is early and strongly upregulated to promote the establishment of DC tolerogenicity [38]. As a result of glucose consumption and glycolysis induction, high amounts of lactate are produced by VitD3-tolDC. Interestingly, lactate has been reported as a novel mechanism producing suppression of T cell proliferation by human autologous tolerogenic dendritic cells (ATDCs) [28]. Along these lines, the metabolic analysis of cells containing *CD115* siRNA exhibited a reversion of the metabolic reprogramming of VitD3-tolDC, showing low glucose consumption, a partial blockage of the PFKFB4 gene expression induction, reduced lactate secretion and low acidification of the medium. Collectively, results highlight CD115 (CSF1R) signaling through CSF1 as one of the key regulators of VitD3-tolDC functions by inducing metabolic reprogramming of VitD3-tolDC towards glycolysis and lactic acid production. Of note, the role of CSF1-CSF1R in the tolerogenicity of VitD3-tolDC is partial and further analysis of potential molecules is required to identify tolerogenic pathways induced in VitD3-tolDC and discover functional biomarkers of these cells.

In this study, we established an optimized method based on ViroB to transfect monocytes with siRNA on day 1 of culture to evaluate their tolerogenic function on VitD3-tolDC. By studying other genes found differentially expressed in VitD3-tolDC compared to mDC, we expect to unravel the tolerogenic signaling of VitD3-tolDC to improve their efficacy for their translation into the clinic.

## 4. Material and Methods

### 4.1. Monocyte Isolation

To obtain monocytes, buffy coat samples from the Banc de Sang i Teixits (Barcelona, Spain) were obtained following the institutional standard operating procedures for blood donation.

First, samples were depleted of CD3^+^ cells using RosetteSep^®^ Human Monocyte Enrichment Cocktail kit (StemCell Technologies, Vancouver, Burnaby, BC, Canada) followed by a ficoll-hypaque (Rafer, Zaragoza, Spain) density gradient separation. After that, isolation of CD14^+^ cells was performed by using EasySep^®^ Human CD14 Positive Selection kit (StemCell) following the manufacturer’s instructions. Finally, viability and cell counting of viable cells were determined by staining (20 min at 4 °C in dark) with 7-amino-actinomycin D (7-AAD) (BD Biosciences, Franklin Lakes, NJ, USA) and phycoerythrin (PE)-conjugated annexin V (Immunotools, Friesoythe, Germany) and the use of PerfectCount microspheres (Cytognos, Salamanca, Spain) for cell quantification. Samples were acquired on a FACSCanto II flow cytometer and analyzed using the FACSDiva software (BD Biosciences).

### 4.2. Generation of VitD3-tolDC

Viable monocytes were cultured at a density of 1 × 10^6^ cells/mL in 24-well plates in IMDM (Thermo Fisher Scientific, Waltham, MA, USA) supplemented with 2% heat-inactivated human AB serum (Sigma-Aldrich, St. Louis, MO, USA), 250 U/mL IL-4 and 200 U/mL granulocyte macrophage colony-stimulating factor (GM-CSF) (both from Peprotech, London, UK) for 6 days at 37 °C and 5% CO_2_ atmosphere. On day 4, culture cell medium and cytokines were refreshed and a maturation cocktail containing 1000 U/mL IL-1β, 1000 U/mL TNF-α (both from Peprotech) and 1 μM prostaglandin-E2 (PGE2) (Pfizer, New York, NY, USA) was added to obtain mDC. For the generation of VitD3-tolDC, monocytes were additionally treated with 1 nM 1α,25-dihydroxyvitamin D3 (Calcitriol, Kern Pharma, Barcelona, Spain) on days 0 and 4. On day 6, cells were harvested by 25 min of accutase incubation at 37 °C to detach the cells from the plate. After washing, cell counts and viability were assessed by flow cytometry as previously mentioned, and phenotype and functional characterization were determined (see below). Dry pellets of each condition with the remaining cells were stored at −80 °C.

### 4.3. Transfection of Monocytes Using HiPerFect Reagent

For the transfection of monocytes on day 5 of culture (after the maturation stimulus), the reverse protocol described by Troegeles et al. [29] was followed and optimized. Briefly, a supernatant of culture was kept and cells were harvested using accutase incubation. After determining the number of viable cells, 1 × 10^6^ cells were resuspended in non-supplemented RPMI (Thermo Fisher Scientific) at a cell density of 1 × 10^6^ cells in 250 µL of culture medium. For the formation of the lipid–siRNA complexes, 3.75 µL of HiPerFect transfectant reagent (Qiagen, Hilden, Germany) were added to 117.5 µL of warm and non-supplemented RPMI medium containing the DY-547-labeled non-targeting siRNA (siGLO, Thermo Scientific, Dharmacon, Illkirch, France). Different volumes of siGLO were tested to determine the optimal siRNA concentration (200 nM, 300 nM, 400 nM, 600 nM, 700 nM or 800 nM) required to achieve a high transfection efficacy of cells. Each lipid and siRNA mix was incubated for 15 min at room temperature in the dark and the tube was gently inverted several times during the incubation to increase the formation of lipid–siGLO complexes. Following the incubation, lipid–siGLO complexes were spinned down and 117.5 µL was transferred to a 24-well plate well. Then, 250 µL of cells (1 million) were pipetted directly into the siRNA complexes and mixed by a gentle swirl of the plate. After a 4 h incubation at 37 °C in the incubator, transfection was stopped by adding 500 µL of previously collected supernatants plus 125 µL IMDM with 10% human AB serum. Finally, cells were incubated for additional 36 h and the phenotype and functionality of the cells were assessed (see below).

### 4.4. Transfection of Monocytes Using Viromer Blue Reagent

To transfect monocytes on day 1, at the beginning of the process of differentiation to VitD3-tolDC, Viromer Blue transfection reagent (ViroB, Lipocalyx, Halle, Germany) was used following manufacturer’s instructions for forward transfection. Briefly, ON-TARGETplus SMARTpool siRNA targeting *CD115* or *CD209* (Dharmacon) was diluted in buffer blue to a final concentration of 2.75 μM. In a separate tube, 2 µL, 1 µL and 0.75 µL of ViroB were diluted with 180 µL, 90 µL and 67.5 µL buffer blue, respectively, and mixed by 3–5 s of vortex. For complexation, 180 µL, 90 µL and 67.5 µL of diluted ViroB were added into 20 µL, 10 µL and 7.5 µL of siRNA, respectively, mixed gently and incubated for 15 min at room temperature. Finally, 200 µL, 100 µL or 75 µL of siRNA-containing polymer vesicles were added to each well containing monocytes on day 1 of culture differentiation. For inducing the downregulation of *CD115* and *CD209* (double knockdown), the working siRNA concentration combined for the 2 genes remained as 2.75 µM (half from each specific gene). In some experiments, two steps of transfection were carried out on day 1 and again on day 4 to ensure the maximum effect of gene silencing during VitD3-tolDC generation and characterization. Cell transfection without siRNA (containing only blue buffer) or with ON-TARGETplus non-targeting control siRNA (Dharmacon) were used as controls to determine unspecific effects of ViroB and siRNA molecules, respectively. In addition, cell transfection with the red transfection indicator, siGLO, was used to analyze transfection efficacy.

### 4.5. Chemical Inhibition of CSF1R

As a complementary control, monocytes were treated on day 0 and day 4 with 4 µM of PLX5622 (kindly provided by MedChemExpress, Mo nMouth Junction, NJ, USA), which inhibits the receptor tyrosine kinase activity of CSF1R (CD115). On day 6, the effect of blocking CD115 cell signaling during monocyte differentiation into VitD3-tolDC was analyzed by phenotype and functional characterization.

### 4.6. CSF1 and IL34 Treatment

To further analyze the role of CD115 signaling during monocyte differentiation, 100 ng/mL of their ligands, CSF1 and IL34 (both from Peprotech), was added independently or in combination to the culture of mDC and tolDC on day 0 and day 4. Phenotype and functional characterization of CSF1- and/or IL34-treated cells were analyzed on day 6.

### 4.7. Phenotype Analysis

Once the cultured cells were harvested on day 6, the expression of the surface molecules CD11c, CD14, CD83, CD86, CD115, CD209 and HLA-DR was determined by flow cytometry. Cells were incubated for 20 min at room temperature in dark with the proper amount of the corresponding monoclonal antibodies (anti-): CD11c phycoerythrin (PE)-Cyanine dye 7 (PE-Cy7), CD14 Violet 450 (V450), CD83 allophycocyanin (APC), CD86 fluorescein isothiocyanate (FITC), CD115 PE, CD209 APC and HLA-DR Violet 500 (V500) (all of them from BD Biosciences, except CD209 APC from BioLegend, San Diego, CA, USA). Samples were acquired on a FACSCanto II flow cytometer and analyzed using the FACSDiva software (BD Biosciences).

### 4.8. Allogeneic Proliferation Assay

Fresh allogeneic PBMCs were obtained from whole blood samples following ficoll-hypaque density gradient separation. Subsequently, cells were resuspended in PBS at concentration of 8 × 10^6^ PBMC/mL and were incubated with 1 mM BD Horizon Violet Proliferation Dye 450 (VPD450) (BD Biosciences) for 12 min at 37 °C in dark. In order to evaluate the ability of the generated DC to stimulate allogeneic PBMC, a proliferation assay was performed in 96-well round bottom plates with co-cultures of 10^5^ allogeneic PBMCs and 5000 DCs (1:20 ratio) or 10,000 DCs (1:10 ratio) in a total volume of 200 μL of IMDM supplemented with 2% human AB serum. Four replicates of each condition were performed. Finally, cells were incubated for 5 days at 37 °C in a 5% CO_2_. Negative and positive proliferative controls were also included by culturing VPD450-PBMC only with supplemented IMDM medium or adding 5 µg/mL phytohemagglutinin (PHA) (Sigma-Aldrich), respectively. On day 5, samples were placed in a FACSCanto II or FACSLyric flow cytometer (BD Biosciences) and the percentage of proliferated cells was analyzed by VPD450 fluorescence dilution.

In some experiments, unlabeled allogeneic PBMC were co-cultured with DC for 4 days. Then, 1 µCi [3H]-thymidine (PerkinElmer, Waltham, MA, USA) was added to each well and cells were incubated for additional 18h. Cells were finally collected using an automated cell harvester (Micro Cell Harvester Skatron) (Tomtec Inc, Hamdem, CT, USA) and [3H]-thymidine incorporation was measured using a 1450 MicroBeta TriLux liquid scintillation counter (Wallac, Turku, Finland). Six replicas for each condition were performed. Results were expressed as mean of counts per minute (cpm) of replicas.

### 4.9. Gene Expression Analysis

Total RNA was isolated from frozen dry pellets samples using the RNeasy Mini Kit (Qiagen) complemented with DNase digestion with the RNase-free DNase set (Qiagen), following instructions provided by the manufacturer. RNA obtained from each condition was immediately quantified in a Nanodrop ND-1000 spectrophotometer (Thermo Fisher Scientific) and retrotranscribed into complementary DNA (cDNA) using the High Capacity cDNA Reverse Transcription Kit (Applied Biosystems, Foster City, CA, USA). Finally, quantitative PCR (qPCR) was carried out to determine the relative expression of *CD209*, *CSF1R* and *PFKFB4* (6-Phosphofructo-2-Kinase/Fructose-2,6-Biphosphatase 4) genes after 50 cycles of cDNA amplification using the TaqMan Gene Expression Master Mix and specific TaqMan Gene Expression Assays (all of them from Applied Biosystems) in a LightCycler 480 System thermocycler (Roche, Basel, Switzerland). The quantitative expression of each gene was calculated based on the 2^−∆Cp^ method and relative expression was assessed using the mean crossing point (Cp) values of the housekeeping genes *Cyclophilin A* (*CYPA*), *TATA-Box Binding Protein* (*TBP*) and *Glyceraldehyde-3-Phosphate Dehydrogenase* (*GAPDH*).

### 4.10. Metabolic Analysis of Culture Supernatants

To analyze metabolism of cells, consumption of glucose, secretion of lactate and pH of supernatants on day 6 of culture of mDC, VitD3-tolDC and VitD3-tolDC treated with siRNA were examined. Glucose and lactate concentrations were determined in an AU5800 platform (Bekman Coulter; Clare, Ireland) using a standard hexokinase method and a lactate oxidase reaction, respectively. For the detection of pH, a direct potentiometry method was used in a Gem Premier 4000 analyzer (Werfen, MA, USA).

### 4.11. Statistical Analysis

Differences between the studied conditions were analyzed using Prism v6.0 software (GraphPad, La Jolla, CA, USA). For comparisons between two groups, a paired Student t-test or a Wilcoxon test were used if the samples were normally distributed or not, respectively. For multiple comparisons, a one-way ANOVA test was used. For the analysis of the relationship between % of HiPerFect transfected cells and RNA concentration, linear regression was performed. Results of all these analyses were expressed as mean ± standard deviation (SD), unless noted otherwise, and the results were considered statistically significant when *p*-value < 0.05.

## Data Availability

Not applicable.

## Supplementary Materials

The following are available online at https://www.mdpi.com/article/10.3390/ijms22147363/s1, Figure S1: Manipulation of cells during HiPerfect transfection on day 4 of culture prevent tolDC generation.
