# Supplementary material for: Transfection of Vitamin D3-Induced Tolerogenic Dendritic Cells for the Silencing of Potential Tolerogenic Genes. Identification of CSF1R-CSF1 Signaling as a Glycolytic Regulator
Source: Int J Mol Sci. 2021 Jul 8;22(14):7363. doi: 10.3390/ijms22147363 (PMC8305050; doi:10.3390/ijms22147363)
Supplement: Supplementary file 1 [file ijms-22-07363-s001.zip › ijms-1255776-supplementary.pdf]

Supplementary Figure 1

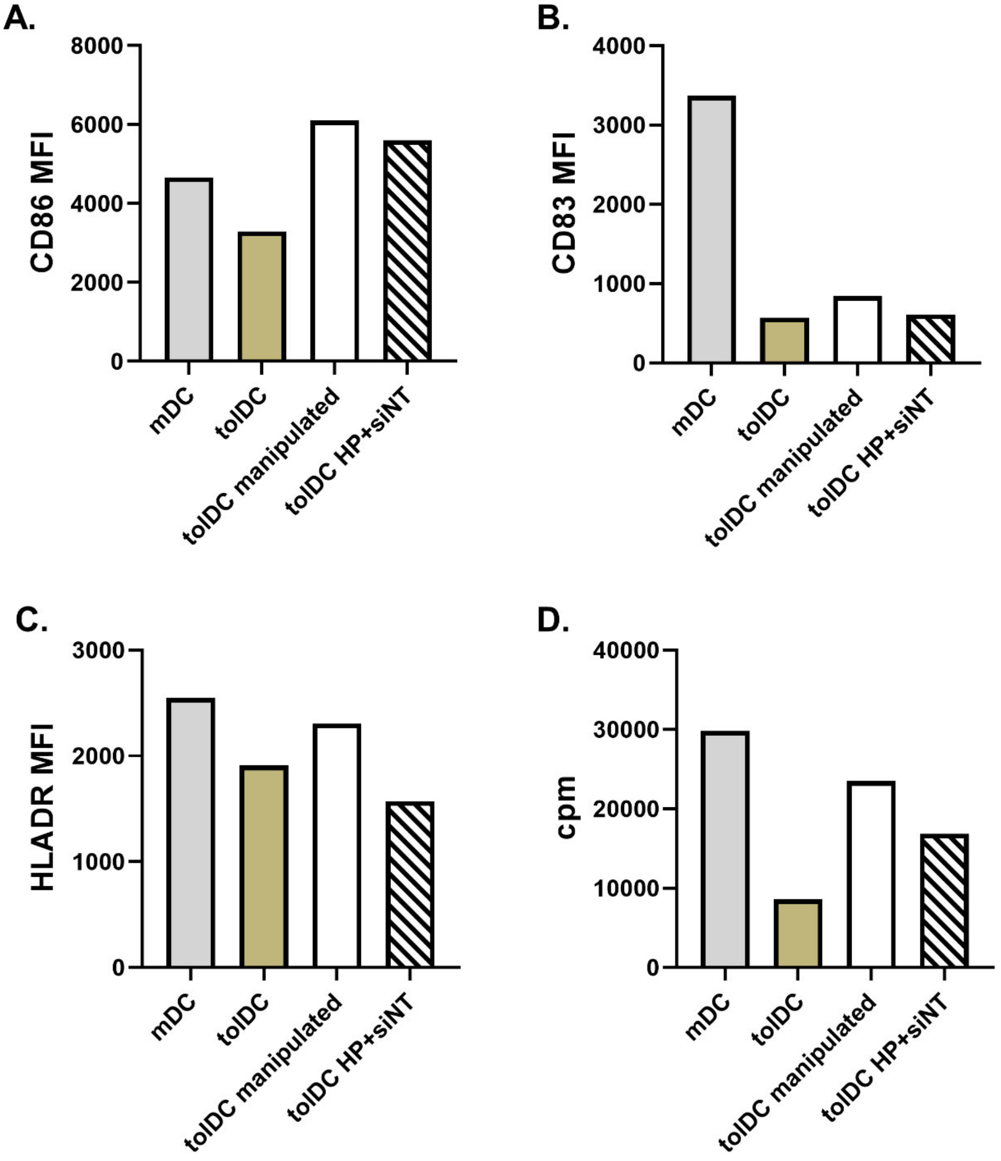

Supplementary Figure S1. **Manipulation of cells during HiPerfect transfection on day 4 of culture prevent tolDC generation.** One representative example of phenotypical (mean fluorescence intensity, MFI, of CD86 (A), CD83 (B) and HLA-DR (C) molecules) and functional characterization (ability to induce allogeneic PBMC proliferation using a ratio of DC/PBMC of 1/20 (C)) of control untreated mDC and VitD3-tolDC, VitD3-tolDC manipulated without using transfection reagents on day 4 of culture and VitD3-tolDC transfected with HiPerfect and non-targeting siRNA on day 4 of culture.
